# Beneficial Effect of Vitamin D on Non-Alcoholic Fatty Liver Disease (NAFLD) Progression in the Zebrafish Model

**DOI:** 10.3390/nu15061362

**Published:** 2023-03-10

**Authors:** Lihi Grinberg, Fadwa Dabbah Assadi, Gideon Baum, Romy Zemel, Ran Tur-Kaspa, Chen Shochat, David Karasik, Marcela V. Karpuj

**Affiliations:** 1 Azrieli Faculty of Medicine, Bar-Ilan University, Safed 1311502, Israel; 2 Felsenstein Medical Research Center, Petah Tikva 49100, Israel; 3 Liver Institute, Rabin Medical Center, Petah Tikva 49100, Israel; 4 Department of Biotechnology Engineering, Braude College, Karmiel 216100, Israel

**Keywords:** NAFLD, vitamin D, zebrafish, dyslipidemia

## Abstract

A major cause of chronic liver disease, cirrhosis, and hepatocellular carcinoma, non-alcoholic fatty liver disease (NAFLD) results from excessive liver fat accumulation. Vitamin D (VitD) plays multiple important roles in diverse physiologic processes. Here, we describe the role of VitD in the complex pathogenesis of NAFLD and explore the possible therapeutic role of VitD supplementation in NAFLD therapy. To compare the effect of VitD to other interventions such as low-calorie diet, we induced NAFLD in young adult zebrafish (*Danio rerio*, AB strain) and monitored the effects of VitD supplementation on the disease course. The zebrafish administered with high-dose VitD (1.25 μg) had significantly reduced liver fat compared to those that received low-dose VitD (0.049 μg) or caloric restriction. Gene expression analysis revealed that VitD downregulated several pathways that may play a role in NAFLD etiology, which affected fatty acid metabolism, vitamins and their cofactors, ethanol oxidation, and glycolysis. The pathway analysis revealed that the cholesterol biosynthesis pathway and the isoprenoid biosynthetic process pathway were significantly upregulated whereas the small molecule catabolic process pathway significantly downregulated following the exposure of NAFLD zebrafish model to high VitD dose. Therefore, our findings suggest the association of novel biochemical pathways with NAFLD and highlight the potential of VitD supplementation to reverse the severity of NAFLD, especially in younger people.

## 1. Introduction

Worldwide, an unbalanced diet and physical inactivity are associated with obesity and metabolic syndrome. Non-alcoholic fatty liver disease (NAFLD) is a multifactorial disorder influenced by genetic and environmental factors [1]. NAFLD is often associated with metabolic syndrome features [2,3], especially cardiometabolic risk factors in obese people with NAFLD (e.g., insulin resistance, dyslipidemia, hypertension, pre-diabetes, and type 2 diabetes) [4,5,6]. The importance of immediately addressing and reducing NAFLD severity recently became more relevant considering the COVID-19 pandemic and the direct correlation between NAFLD and COVID-19 severity demonstrated by metanalyses worldwide [7,8]. NAFLD is characterized by pathological lipid accumulation in the liver within hepatocyte cytoplasm in the absence of excessive alcohol consumption. NAFLD develops when hepatic fatty acid availability from the plasma and de novo synthesis exceeds hepatic fatty acid disposal by oxidation and triglyceride export. Therefore, the intrahepatic triglyceride accumulation (i.e., steatosis, the hallmark feature of NAFLD) results from an imbalance between the complex molecular pathways of lipid metabolism [1].

In recent years, NAFLD has become a major public health problem due to its high prevalence and because it has the potential to progress to more severe liver diseases [9]. Evidence from human epidemiological studies suggested that vitamin D (VitD) may play a role in NAFLD pathogenesis [4,10,11,12]. For example, VitD metabolic pathway polymorphisms are associated with histological severity of pediatric NAFLD [13]. VitD and its metabolites are crucial parts of the endocrine system that control whole-body calcium and phosphorus homeostasis [14]. Furthermore, VitD exerts pleiotropic effects; it is involved in immunomodulation, has anti-inflammatory, anti-apoptotic, and anti-fibrotic roles, and is involved as a natural anti-viral mediator [15]. Additionally, VitD modulated lipid metabolism [16] and liver lipid metabolism in diabetic rat models [17]. Nevertheless, there is some controversy in the literature. Some studies have found no correlation between VitD and NAFLD, and the levels of VitD could neither determine nor predict the severity of the diseases [18,19,20,21,22,23]. Moreover, it was demonstrated that there is no causal association between vitamin D and NAFLD using a bi-directional Mendelian randomization approach in a Chinese population [23,24]. Finally, evidence supports the hypothesis of the potential benefits of VitD supplementation in selected populations of NAFLD patients, such as those with shorter disease duration and mild to moderate liver damage [25].

As lifestyle interventions such as diet alterations to induce weight loss and physical activities are beneficial for improving NAFLD [26,27], but are not always feasible, the demonstration of a causal role of hypovitaminosis D in NAFLD etiology could have important therapeutic implications [28,29]. NAFLD diagnosis should preferably be based on the histological analysis of liver biopsies by measuring lipid accumulation using lipid staining [30,31]. However, liver biopsies in humans are invasive and often painful. Therefore, etiology studies, especially intrahepatic change and molecular mechanistic studies, must be performed using animal models.

The zebrafish (*Danio rerio*) has been widely recognized as a feasible model for several human diseases for over two decades [32,33]. NAFLD can be established in small fish such as zebrafish or medaka [34,35,36] via high caloric intake, i.e., overfeeding. Similar to humans with NAFLD, zebrafish with NAFLD exhibit increased body mass index (BMI) and hypertriglyceridemia, which progress to hepatic steatosis [37,38]. Therefore, zebrafish can be used to identify putative pharmacological targets and test novel strategies for treating NAFLD.

Although limited research has been conducted on fish [39] (especially adult fish [31]), the function of their endocrine system and identified metabolites appear similar to those of terrestrial vertebrates [40].

In this study, our results indicated that the addition of high-dose VitD to fish food significantly decreased fat excess in the liver. These results were in accordance with the recent findings by Knuth et al. [41] and might explain the fact that other studies did not find a benefit from VitD since they did not explore the possibility of high-dose VitD [42]. Notably, these results might be relevant for mitigating and treating COVID-19 with an additional boost of high-dose VitD. This hypothesis is strengthened by meta-analysis studies demonstrating the reverse correlation between high-dose VitD and COVID-19 infectivity [43,44,45].

However, no clear mechanism explains this phenomenon. Here, we demonstrate that VitD supplementation activated genes in several pathways. The biochemical processes affected by high-dose VitD included the pathways for fatty acid metabolism, the metabolism of other vitamins and their cofactors, and ethanol oxidation and glycolysis. These pathways may all play a role in NAFLD etiology.

## 2. Materials and Methods

### 2.1. Fish Husbandry

Zebrafish (AB strain, short fin) were raised in closed recirculating housing systems at 28 °C, pH 7.5, under a photoperiod regime of 14 h light 10 h dark cycle and maintained according to IACUC/national committee’s approval for the Bar Ilan University zebrafish facility (number 023_b7241_24), as described [46]. 

### 2.2. Zebrafish NAFLD Model

The zebrafish NAFLD model was established by overfeeding adult fish with Tetramin fish food (Tetra, Blacksburg, VA, USA) for two months. To generate NAFLD, we used the protocol for a diet-induced obesity model described in Oka et al. [37]. Three-month-post-fertilization (mpf)-old male and female zebrafish were fed with a high caloric diet composed of Tetramin, three meals/day, for two months. Each experimental group was housed together at a density of 10 fish per 3L tank and fed together ad libitum. The establishment of NAFLD in zebrafish was determined by measuring fat levels in liver sections stained with Oil Red O (ORO).

### 2.3. VitD Supplementation in NAFLD Zebrafish Food

Several feeding regimens were applied to NAFLD zebrafish (all shown per 3L tank with 10 fish): 3 meals/day (no VitD added), 3 meals/day with 0.049 µg VitD per meal (Cholecalciferol, Sigma, St. Louis, MO, USA) (low VitD), 3 meals/day with 1.25 µg VitD per meal (high VitD), one meal/day (caloric restriction). This VitD is the non-active form, which is converted in zebrafish to its active form, 1,25(OH)_2_D_3_. Non-NAFLD zebrafish were fed 2 meals/day (control) or 2 meals/day with traces of ethanol (vehicle control). In each meal fish received 300 mg of food. Since Tetramin fish food contains 50 µg/kg of VitD (according to manufacturer’s data), all feeding groups received at least 0.015 µg VitD in each meal. Since we wanted to add VitD to fish food, and since it is not soluble in water, we first dissolved it in ethanol (99%). We then mixed it with 400 g Tetramin as suggested in [47], based on the requirements for micronutrient intake for zebrafish and considering dose–response analysis suggested by Fleming et al. [48]. Further, this admixed Tetramin VitD was grounded and filtered with a 700 nm strainer to obtain small bite-sized pieces, and dried overnight at room temperature to remove ethanol traces. To rule out any influence of the ethanol used to dissolve VitD, we fed fish with Tetramin mixed only with ethanol and dried overnight (preparation was the same as VitD supplemented food but without VitD), referred to as “vehicle control” group above.

### 2.4. Euthanasia and Dissection

At the end of the experiment, fish were euthanized and their gender, weight, and length were measured [49]. Fish were euthanized by immersion in 200–300 mg/L Tricaine methanesulfonate (MS222, Sigma, St. Louis, MO, USA) for 10 min. Whole-body length and weight were measured immediately after anesthetization. Zebrafish length was measured from the head to the end of the body (without the tail) with a ruler. Weight was recorded using the Sartorius ED124S scale. BMI was calculated by dividing the body weight (g) by the square of the body length (cm) according to Oka et al. [37]. Gonads were dissected for sex determination.

### 2.5. Hematoxylin and Eosin (H&E) Staining

Liver tissues were collected from zebrafish by surgical manipulation under a stereoscopic microscope (M80; Leica Microsystems, Wetzlar, Germany). Livers were embedded in optimum cutting temperature compound, OCT (Tissue-Tek, Elkhart, PA, USA), following rapid freezing on dry ice. Fresh frozen samples were sliced to 8-microns-thick slices using Cryostat (CM3050 S; Leica Biosystems, Dublin, Ireland) at −20 °C. Slides were fixed with 95% ethanol, washed in water, and dipped in Harris hematoxylin (Leica, Cambridge, UK) for 2 min. Next, they were washed in ethanol, dipped in eosin solution for 2 min, washed with ethanol, dipped in xylene for 1 min, dried for 10 min, and mounted with xylene-based mounting medium. Photographs were obtained by Axio Cam ICc3 (Zeiss, Thornwood, NY, USA) for tissue morphology analysis.

### 2.6. Oil Red O (ORO) Staining

ORO staining of hepatocytes indicates the presence of intracellular lipids. Frozen liver sections were fixed in 4% paraformaldehyde and rinsed with distilled water. The sections were immersed in 60% isopropanol and then in a solution of Oil Red O (Sigma, St. Louis, MO) for 15 min at room temperature [50]. Light microscopy was carried using Axio Imager 2 (Zeiss, Oberkochen, Germany) and images were obtained using Axio Cam ICc3. Image analysis software (ImageJ/Fiji, Bethesda, MD, USA) was used to measure the ORO-positive areas in tissue images. The liver lipid level (%) was quantified as a ratio of ORO stained area to the total area of hepatic tissue. Results were analyzed with a two-way analysis of variance test (ANOVA) [51]. Statistical significance was set at *p* < 0.05. 

### 2.7. RNA Extraction 

Total RNA was extracted from zebrafish liver and purified by QIAGEN-RNeasy^®^ Fibrous Tissue Mini Kit (Qiagen, Venlo, Netherlands). RNA quality was assessed by NanoDrop 1000 (Thermo Fisher, Waltham, MA, USA). 

### 2.8. RNA Sequencing and Data Analysis 

For RNA sequencing, we pooled RNA extracted from 3 male fish representing nutrition groups (2 replicates for each group, n = 6): no VitD added, low VitD, high VitD, and vehicle control. cDNA library construction and next-generation sequencing were carried out at the Genome Technology Center at Bar-Ilan University/Faculty of Medicine. Quality control of initial RNA material was assessed using the RNA pico kit on the Agilent 2100 Bioanalyzer, and only samples with RNA integrity number (*RIN*) above seven were included in this study. mRNA libraries were generated using NEBNext library reagent (Cat # E7420; New England Biolabs, Ipswich, MA, USA) according to NEB’s protocols [52], with starting material of 100 ng of total RNA. The Illumina Hi-Seq^®^ 2500 platform (Illumina, La Jolla, CA) was used to generate 61 base pair-long single-end reads. Libraries were applied to an Illumina flow cell (four samples per lane) at a concentration of 10 pM. We obtained an average of 41 million reads per sample (range 35 M–50 M). Trimmed reads were generated by Illumina’s cloud tool Basespace^®^. Pre- and post-alignment quality control, genome alignment, and differential gene expression were analyzed using Partek^®^ Genomic Flow software (Partek, St. Louis, MO, USA). Danio rerio (Zebrafish)-DanRer10 was utilized for alignment using Star2.4.1d aligner index. Generated counts were normalized using Reads Per Kilobase of transcript, per Million mapped reads (RPKM). 

### 2.9. Bioinformatic Analysis and Annotation

In the RNA-seq experiment, we compared the gene expression between NAFLD (no VitD added) and other diet groups (high and low VitD and vehicle control groups). Student’s *t*-test was performed to assess the significance of the differences between groups. Genes were considered differentially expressed if the false discovery rate (FDR) was less than 0.05 and there was at least 2-fold difference in expression. To explore the functional features of the identified genes, we used the online tool ToppGene Suite ([53], toppgene.cchmc.org (accessed on 1 March 2023)), which quantifies functional enrichment analysis including Gene Ontology term, KEGG canonical pathways analysis, and *Ingenuity* Pathway Analysis (IPA https://digitalinsights.qiagen.com/ (accessed on 1 March 2023)

### 2.10. RT-qPCR and Data Analysis

Since false-positive results are typical in cDNA microarrays, we sought to confirm gene expression differences by RT-qPCR assay using the same RNA samples that were used in the RNA sequencing. For this, total RNA was extracted as described above. cDNA was synthesized using a High-Capacity cDNA Reverse Transcription Kit and RT-qPCR (Biosystems, Foster City, CA, USA). All procedures were performed according to the manufacturer’s guidelines. 

Real-time RT-PCR assays were performed in ViiA™ 7 Dx Real-Time PCR Instrument (Life Technologies Corporation, Gaithersburg, MD), using FastStart Universal SYBR Green Master with FastStart Universal Probe Master (Roche, Mannheim, Germany; see Supplementary Table S2 for RT-qPCR primer sequences). PCR cycling conditions were as follows: an initial denaturation step at 95 °C for 10 min, then 15 s at 95 °C, and 1 min at 60 °C for 40 cycles. *EF1α* (*eef1a1l1*, accession no. NM_131263) was used as a reference (“housekeeping”) gene. Moreover, we compared cyp24a1 gene expression among the experiment groups. CYP24A1 functions in VitD target tissues to degrade 1,25(OH)_2_D_3_. Therefore, the regulation of this enzyme’s expression is a primary determinant of the overall biological activity of 1,25(OH)_2_D_3_ within tissue [54]. Experiments were conducted in biological and technical triplicates. Results of individual RT-qPCRs were analyzed using the delta delta C_(T)_ equation as described [55]. Results were analyzed with a two-way analysis of variance test (ANOVA) [51]. Graphs were created using Graphpad Prism V6.0 (Graphpad Prism software, San Diego, CA). Student’s *t*-test was performed to compare means ± standard deviation between groups. Statistical significance was set at *p* < 0.05. 

### 2.11. Human Cell Experiments: HepG2 Cell Culture

We analyzed the effect of VitD (Cholecalciferol) and 1,25(OH)_2_D_3_ on lipid accumulation in HepG2 cells, a human liver cancer cell line widely used to model NAFLD in vitro. HepG2 cells purchased from ATCC (ATCC^®^ HB-8065™) were cultured in EMEM (MEM-Eagle Minimum Essential Medium with Earle’s Salts) supplemented with 15% fetal bovine serum and Penicillin-Streptomycin-Amphotericin B Solution (Biological Industries, Beit Haemek, Israel) at 37 °C in 5% CO_2_. At 60–80% confluence, cultures were routinely sub-cultured using 0.05% Trypsin-EDTA (Biological Industries). 

### 2.12. Steatosis Induction in HepG2 Cells 

For the experiments, cells were plated in 96- or 24-well plates with a medium supplemented with 10% charcoal-stripped fetal bovine serum (Biological Industries). After 24 h, the medium was supplemented with incremental doses up to the non-toxic level of 168 nM 1,25(OH)_2_D_3_ (Sigma, St. Louis, MO, USA). Similar volumes of vehicle (EtOH) were used as controls. Two hours later, the culture medium was replaced with a steatosis medium supplemented with the same concentrations of 1,25(OH)_2_D_3_. Cells were cultured for two more days. Preparation of the steatosis medium was as follows: each free fatty acid (FFA) sodium salt was dissolved in Isopropanol to 200mM, diluted in EMEM containing 10% charcoal-stripped fetal bovine serum and 1% (*w/v*) FFA-free bovine serum albumin (BSA) to final concentration of 100 µM oleic acid and 200 µM palmitic acid (Sigma, St. Louis, MO, USA) and was incubated in 37 °C for 2 h with shaking. 

### 2.13. Assessment of Lipid Accumulation in HepG2 Cells by Oil Red O Staining

The cells were washed two times with phosphate-buffered saline (PBS) and fixed with 1 mL 4% paraformaldehyde (PFA) for 1 h at room temperature followed by 2 washes with ddH_2_O and a 5 min wash with 60% isopropanol. The cells were left to dry completely and then stained for 30 min with 50 μL of working solution of Oil Red O (5% ORO in isopropanol mixed before use with H_2_O at 60%:40% ratio and filtered) at room temperature followed by 2 washes with H_2_O. The staining was assessed under a microscope. To measure the level of steatosis semi-quantitatively, the cells were washed 3 times for 5 min each with 60% isopropanol and Oil Red O stain was extracted with 100 µL 100% isopropanol for 5 min. The absorbance was measured at 492 nm using an InfiniteM1000 photometer (Tecan Trading AG, Männedorf, Switzerland).

### 2.14. Measurement of the Viability of HepG2 Cells following Treatment 

CellTiter-Fluor™ Assay was used; it measures protease activity within live cells and serves as a marker of cell viability. The assay was performed according to the manufacturer’s protocol (Promega, Madison, WI, USA), on 96-well clear-bottom, opaque-walled tissue culture plates. 

## 3. Results

**Figure 1:**
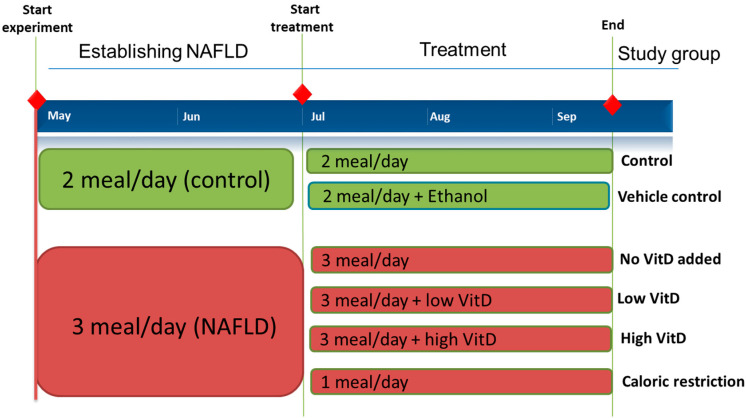
Diet and VitD supplementation effect on NAFLD in zebrafish: timeline of experiment. Establishing NAFLD in zebrafish was achieved by feeding 3 meals a day for 2 months while control group were fed twice a day. Afterwards, fish were divided into 6 study groups and treatment was applied for 3 months.

Adult zebrafish were fed a high-fat diet to induce NAFLD and to determine whether they developed similar hepatic manifestations to humans. Young adult zebrafish (3 months post-fertilization (mpf), age of beginning of reproduction) were overfed by administering three meals a day for 2 months (Figure 1). Hematoxylin–eosin (H&E) and Oil Red O (ORO) staining of liver tissue from the overfed zebrafish revealed the presence of hepatic steatosis, a higher percentage of liver fat, and large vacuoles in the cytoplasm, confirming the NAFLD zebrafish phenotype (Supplementary Figure S1). In a preliminary experiment, the NAFLD zebrafish received low-VitD supplements or a caloric restriction diet to discern the efficacy of VitD. The BMI measurements revealed no difference between the treatment groups (Supplementary Figure S2) or the sexes (data not shown). ORO staining revealed lower liver fat levels in the low-VitD-supplemented fish (n = 24) in comparison to the unsupplemented fish (n = 66) (Supplementary Figure S1). Furthermore, the caloric restriction group had a similar fat level to the low-VitD and control groups (Supplementary Figure S2).

**Figure 2:**
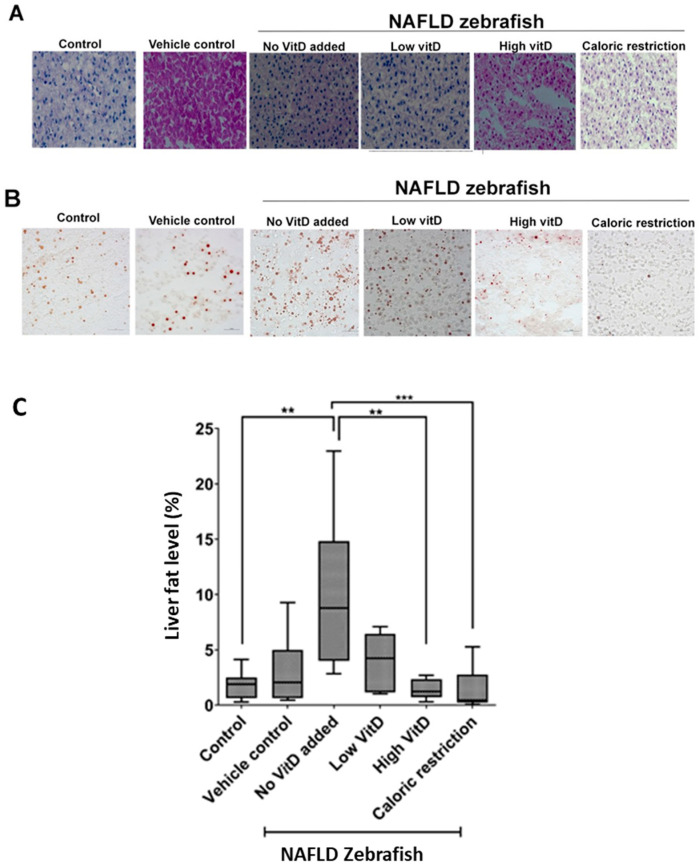
Liver morphology and fat level in NAFLD zebrafish supplemented with VitD. Histology staining of liver sections was performed in the following groups: control (normal feeding; 2 meal/day/3L tank), vehicle control (2 meal/day/3L tank with ethanol addition), no VitD added (3 meal/day/3L tank), low VitD (3 meal/day/3L tank with 0.049 µg VitD per meal), high VitD (3 meal/day/3L tank with 1.25 µg VitD per meal), caloric restriction (1 meal/day/3L tank). (**A**) Liver morphology by hematoxylin and eosin staining. (**B**) Representative images of lipid content in the liver measured by ORO staining. Magnification ×40, scale bar 20 µm. (**C**) For each group, images of ORO-stained area were used to calculate the fat level in the liver. ** *p* < 0.05, *** *p*< 0.001.

Based on these results, we subsequently focused on nutritional interventions targeting NAFLD using high and low VitD dosages (Figure 1) to alleviate NAFLD. H&E staining of the liver tissue revealed the presence of hepatic steatosis in the NAFLD fish fed a high-fat diet without VitD supplementation (n = 8) (Figure 2A). ORO staining demonstrated decreased (*p* < 0.05) fat levels in the NAFLD fish fed a high-fat diet with high VitD (n = 9) in comparison to NAFLD fish fed a high-fat diet without VitD supplementation. In fish fed a high-fat diet with low VitD (n = 6), the apparent decrease in fat level was not significant (Figure 2B,C). Additionally, the fish with caloric restriction (n = 8) had significantly reduced (*p* < 0.001) fat levels (Figure 2C) as previously demonstrated by Oka et al. [37].

The differences in fat levels between the vehicle control group (n = 7) and the control group (n = 7) were not significant. This implied that trace amounts of ethanol (if any) in the VitD-supplemented groups did not influence fat accumulation in the liver (Figure 2C).

### 3.1. Gene Expression Analysis Revealed Activation of Pathways Involved in VitD Metabolism and NAFLD

**Figure 3:**
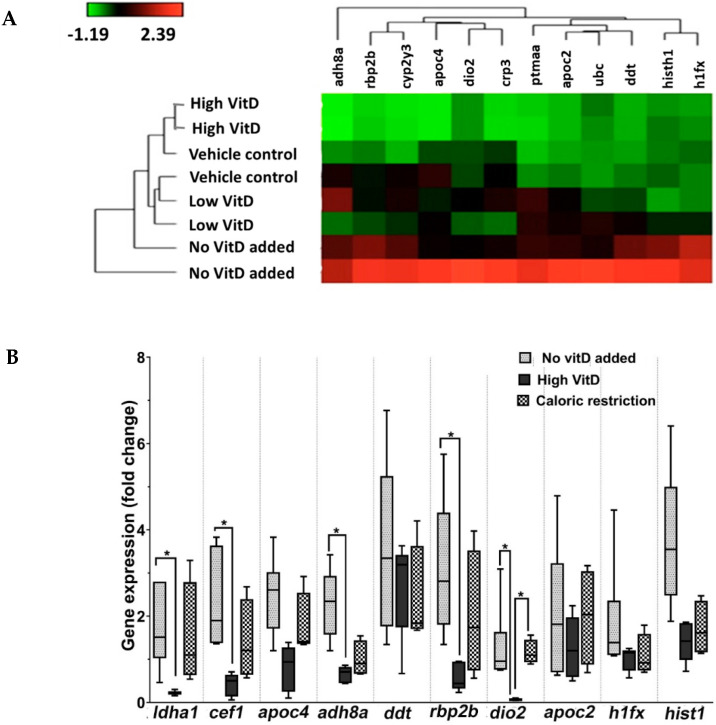
RNA sequencing and validation of the top genes by RT-qPCR in NAFLD zebrafish supplemented with VitD. (**A**) Heat map of top differentially expressed protein-coding genes. The expression of 12 genes was downregulated significantly in high VitD-supplemented as compared to NAFLD group (FDR-adjusted *p* < 0.05). (**B**) Gene expression by RT-qPCR in NAFLD, NAFLD plus high VitD, and caloric restriction groups. * *p* < 0.05.

Transcriptomic analysis was performed to compare differential gene expression between the following nutrition groups: no VitD supplementation, low VitD, high VitD, and vehicle control (n = 6 per group, males only). According to the pre-specified criteria (false discovery rate (FDR) < 0.05 and two-fold difference in expression), expression was lower for 12 genes in the high-VitD group as compared to the no-VitD group (Figure 3A). Bioinformatic analysis using the ToppGene Suite [53] revealed that these genes code for proteins belonging to the hemoglobin subunits, carboxylesterases, fatty-acid-binding proteins, apolipoproteins, and histones. The top molecular functions included oxygen transporter activity, hemoglobin/oxygen/iron ion binding, and oxidoreductase activity. The enriched biological processes were the oxygen transport, oxidation–reduction, and carboxylic acid metabolic processes. The top 12 genes belonged to Reactome pathways such as signaling by retinoic acid, lipid digestion/mobilization/transport, and biological oxidation (Supplementary Table S3).

The Metascape pathway analysis tool [56] of significantly upregulated genes in the transcriptomic analysis (Supplementary Table S1) indicates the effect on two pathways (Supplementary Figure S1a). The highest impact (Log(q-value) of −12.9) was on the cholesterol biosynthesis pathway (WikiPathways WP1387) represented by the following genes: dhcr7, cyp51, sc5d, mvda, fdps, hmgcra, fdft1, lss, sqlea, ebp, cyp7a1, srebf2, cyp24a1, gch2, asah2, sult1st1, gatm, acsl1b, pltp, slc7a3a, enpp7.1, cpox, and mid1ip1l. The second pathway that was significantly impacted (Log(q-value) of −2.4) was the isoprenoid biosynthetic process pathway (GO:0008299). The genes that were significantly increased following high VitD exposure, belonging to this pathway, are *mvda*, *fdps*, *hmgcra*, *lss*, *fdft1*, *acsl1b*, *slc7a3a*, *asah2*, and *enpp7.1*. Interestingly, the small molecule catabolic process pathway (GO:0044282) was the only one that was significantly downregulated (Log(q-value) of −2.7). The genes that were downregulated that belong to this pathway are the following: *hgd*, *adh8a*, *aldh4a1*, *amdhd1*, *abhd3*, *urad*, *miox*, *oat*, *uraha*, *lpin1a*, *aldh1l1*, *me1*, *elovl6*, *hacd2*, *bcat2*, *cyp39a1*, *cyp2ae1*, *acsbg1*, and *acad8*. All of these genes may play a role in NAFLD etiology, impacting the metabolism of fatty acids, vitamins and their cofactors, ethanol oxidation, and glycolysis. These findings point to the association of novel biochemical pathways to NAFLD, and emphasize the potential for VitD supplementation to reverse the severity of disease, especially in younger people.

### 3.2. RT-qPCR Validation of RNA Sequencing

We analyzed the expression of 10 top genes by RT-qPCR using the ViiA7 real-time PCR system. The genes were *ldh1*, *cef1*, *apoc2*, *apoc4*, *adh8a*, *ddt*, *rbp2b*, *dio2*, *h1fx*, and *hist1* (Figure 3B). We also tested *cyp24a1*, whose expression is a marker of activated VitD [1,25(OH)_2_D_3_] biological activity. The mRNA expression levels were compared between the following groups: no VitD, high VitD, vehicle control, and caloric restriction.

The results demonstrated gene expression changes concordantly with those observed following microarray. The RT-qPCR analysis revealed downregulated gene expression in the high-VitD group compared to the no-VitD and even caloric restriction groups (Figure 3B), confirming the RNA sequencing (RNA-seq) results. Although six of the ten studied genes were nominally significant, all demonstrated the same direction of change. As expected, *cyp24a1* gene expression was highest in the high-VitD group (*p* < 0.05), but not in the low-VitD group (Supplementary Figure S3).

### 3.3. Pathway Analysis

We used the Metascape analysis online tool at https://metascape.org/gp/index.html#/main/step1 (accessed on 1 March 2023) [56] to perform a pathway analysis, by placing the entire list of upregulated list of genes in Supplementary Table S1, setting both the input and the analysis setting to the D. rerio option in step 2, and asked for the express analysis graphs in step 3. Thereafter, we accepted only the pathways that had a Log(q-value) value lower than −2 since these are the genes that were recognized as statistically meaningful genes. 

### 3.4. Human Cell Experiments

**Figure 4:**
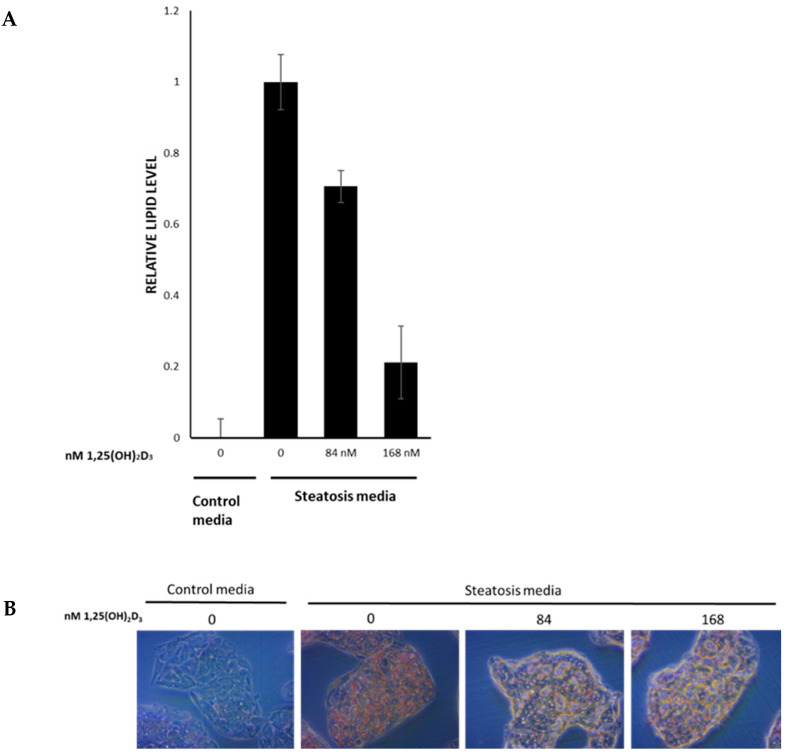
The effect of 1,25(OH)_2_D_3_ on lipid accumulation in HepG2 cells. (**A**) Oil Red O staining of HepG2 cells treated with steatosis medium with different concentrations of 1,25(OH)_2_D_3_. (**B**) Relative measurement of Oil Red O levels extracted from HepG2 cells treated with steatosis medium with different concentrations of 1,25(OH)_2_D_3_. Error bars represent standard error of the mean. n = 3 for VitD treatments and n = 4 for controls.

As opposed to zebrafish cells, human cells cannot convert the inactive form of VitD to its active form [1,25(OH)_2_D_3_]. Therefore, incubating HepG2 cells with the inactive VitD did not affect lipid accumulation in the ORO staining assay (data not shown). However, 1,25(OH)_2_D_3_ exposure significantly inhibited lipid accumulation in the HepG2 cells (Figure 4). The inhibition was concentration-dependent, with maximal inhibition at 168 nM 1,25(OH)_2_D_3_ (Figure 4B). The effect of the treatment on cell viability was assessed by the CellTiter-Fluor assay, where 168 nM 1,25(OH)_2_D_3_ (the highest non-toxic concentration tested) and the corresponding amount of ethanol (the solvent of 1,25(OH)_2_D_3_) did not affect cell viability. Moreover, the amount of ethanol used had no effect on lipid accumulation (Figure 4A).

## 4. Discussion

NAFLD is rapidly becoming the most common liver disease worldwide, where it is prevalent in 20–30% of the general Western population. NAFLD results from excessive liver fat accumulation in the absence of alcohol misuse. Oka et al. demonstrated that fat accumulation was decreased in NAFLD fish after 14-day caloric restriction [37]. Moreover, several drugs that reduce glucose levels (e.g., pioglitazone, glucagon-like peptide 1 (GLP-1), receptor agonists, sodium/glucose co-transporter 2 (SGLT-2) inhibitors), antioxidants (e.g., vitamin E), statins or other lipid-lowering agents, and bile and non-bile acid farnesoid X-activated receptor (FXR) agonists have been tested [57]. However, there are no clear pharmacological strategies to date for treating NAFLD [58]. Therefore, this study was motivated by the search for mitigating agents. Physical activity and nutrition (low-calorie diet) reduce fat accumulation in the liver. NAFLD and low VitD levels are often detected together; hence, VitD deficiency is thought to be involved in the complex pathogenesis of NAFLD [59]. Although there are indications in the literature that low levels of VitD in NAFLD patients might not be the cause of the disease and cannot predict its severity [18,19,21,23,24], we were nonetheless interested in exploring the option of supplementation with high levels of VitD. We hypothesized that since relatively low levels of VitD supplementation were not enough for these patients, the dose of VitD supplementation should be higher. In this study, we successfully induced NAFLD in a zebrafish model (Figure 2C). We demonstrated for the first time that VitD supplementation exerted a statistically significant effect on reversing hepatic fat accumulation in a fish model of NAFLD (Figure 2C). It is important to note that the effect of high-dose VitD was similar to that of a calorie restriction regimen.

To assess the relevance of the results obtained from the NAFLD zebrafish model to human health, we analyzed the effect of the inactive form of VitD and its active form 1,25(OH)_2_D_3_ on lipid accumulation in HepG2 cells, a human liver cancer cell line that is widely used to model NAFLD in vitro [60]. The fact that 1,25(OH)_2_D_3_ significantly inhibited lipid accumulation in cells cultured in a steatosis-inducing medium (Figure 4) corroborated the involvement of the genes and metabolic pathways identified in the zebrafish model (Figure 3 and Supplementary Tables S1 and S3) and the idea that these pathways might be significant targets for preventing and treating NAFLD in humans. Moreover, we demonstrated this specific effect as the inactive form of VitD did not affect lipid accumulation in HepG2 cells.

We also observed a dose-dependent response to VitD supplementation in the zebrafish, with a lower dose exerting only a minor effect on fat accumulation in the liver (Figure 2 and Figure 4). Furthermore, gene expression analysis demonstrated that the administration of VitD (especially in high doses) activated genes in several pathways such as those for fatty acid metabolism, vitamins and cofactors, ethanol oxidation, and glycolysis, all of which may play a role in NAFLD etiology (Supplementary Tables S1 and S3). Similar to other organisms, high doses of VitD might be toxic to zebrafish; however, VitD undergoes detoxification and enterohepatic recirculation with bile acids, then the inactivation products are excreted in bile [31].

In addition, we compared *cyp24a1* expression among the experiment groups and determined that it was highest in the high-VitD-supplemented group (Supplementary Figure S3). As CYP24A1 functions in VitD target tissues to degrade 1,25(OH)_2_D_3_ [54], this finding supports the primary role of VitD in liver tissue. Of the top genes downregulated in the high-VitD group (Figure 3A), ten were tested by RT-qPCR, of which five had significantly decreased expression (Figure 3B). The significantly downregulated genes included the *LDHA* gene, which encodes the A subunit of lactate dehydrogenase (EC 1.1.1.27), an enzyme that catalyzes lactate and pyruvate interconversion. A mutation in the *LHDA* gene causes the metabolic disorder glycogen storage disease 11 (GSD11, OMIM #612933). A homolog to yeast CDC5-like sequence, a Myb-related nuclear protein, the *CEF1* (*CDC5L*) gene is localized in nuclear speckles, is required for pre-mRNA splicing, and is essential for G2–M progression in the cell cycle. The alcohol dehydrogenase genes *ADH8A* and *ADH8B* are partially conserved in zebrafish. These alcohol dehydrogenases share common ancestry with mammalian class I, II, IV, and V alcohol dehydrogenase genes but have distinct functional characteristics [61]. ADH8A bio-transforms longer-chain primary alcohols (≥5 carbons) and S-(hydroxymethyl)glutathione. The retinol-binding protein 2b gene (*RBP2B*) encodes an abundant protein in the small intestinal epithelium with a lipid-binding function. RBP2B participates in vitamin A uptake and intracellular metabolism. Finally, the iodothyronine deiodinase 2 gene (*DIO2*) is widely expressed, including in the thyroid, placenta, pituitary, and the brain. DIO2 is responsible for the local production of triiodothyronine and is thereby crucial in influencing thyroid hormone action in these tissues.

We observed the downregulated expression of the abovementioned genes in NAFLD zebrafish with high VitD supplementation as compared to no-VitD and calorically restricted zebrafish. The involvement of the downregulated genes in metabolic pathways, e.g., fatty acid metabolism or glycolysis, complied with the postulated etiological mechanisms of NAFLD [62]. In turn, the novel molecular pathways illuminated important targets for NAFLD drug treatment or biomarkers for early diagnosis. In our study, the most significantly upregulated genes are involved in the following pathways: retinoic acid signaling, lipid digestion/mobilization/transport, and biological oxidation.

In accordance with previous studies, our results indicated that zebrafish represent an attractive model to study the regulatory mechanisms involved in NAFLD [63] and obesity [41]. The advantage of zebrafish as a model, which explains their preferred use in studies similar to ours, is that it is a vertebrate with conserved liver morphology and well-conserved metabolic physiological mechanisms. Recently, it was demonstrated that dietary components such as high cholesterol, high fructose, and extra feeding resulted in varying degrees of steatosis. One study indicated the pathways of interest that might be involved in NAFLD; however, that study was conducted in larvae [39].

In this study, we focused on adult zebrafish (3 mpf) and quantified the amount of neutral fat in zebrafish liver with ORO staining, a gold-standard method for diagnosing NAFLD in humans via liver biopsies [30,31]. In particular, energy homeostasis regulation is conserved in zebrafish. Therefore, the zebrafish has become the model of choice for identifying the genes or drugs regulating lipid metabolism [64]. New drugs have been tested in zebrafish for decades [65,66]. Specifically for this work, the cholecalciferol pathway is conserved in adult zebrafish [67]; similar to other vertebrates, VitD is metabolized to 25-hydroxycholecalciferol and then to 1,25-dihydroxycholecalciferol in zebrafish liver [68].

We used AB strain zebrafish in this study as it is widely used by the zebrafish research community, especially to study metabolic and obesity-related processes [64], which renders our study replicable. Our choice of a generic strain is important, as some studies suggested that gene expression might differ according to strain [69]. As NAFLD affects even younger people [62], the fish we used were relatively young: we began overfeeding at age 90 days and started treatments at age 5 months. We acknowledge that the response to caloric restriction might differ in aged fish.

In our experiments, we overfed adult zebrafish with normal diet [64], which includes bioavailable VitD. However, we added cholecalciferol as a supplement to mimic the vitamin additives humans receive in addition to their regular VitD-replete diet. Furthermore, we fed the fish without adding specific fat supplements, which might have triggered certain metabolic pathways. In our study, there was no difference in the BMI measurements between treatment groups. Notably, we were able to establish a NAFLD-like phenotype in the fish in the absence of general obesity even with a potential obesogenic diet. This non-obese NAFLD is a phenomenon that has been described in humans [70], which indicates the potential of NAFLD interventions targeting liver metabolism and not the generalized metabolic disease.

The exploratory RNA-seq experiments (Figure 3A) included only male zebrafish in all tested groups, as our pilot experiments did not identify sex-related differences in the response to overfeeding and VitD supplementation. Furthermore, it was suggested that female zebrafish react similarly to diet-induced obesity compared to male zebrafish in terms of BMI [37,71]. However, others reported sex-specific gene expression in zebrafish livers [69]. Therefore, to prevent a possible artifact, we analyzed a subset of male-only fish in the RT-qPCR sample and the results were in agreement with the RNA-seq male sample (Figure 3B). While this study focused on liver gene expression, other gastrointestinal tract organs should be examined. Nevertheless, independent studies demonstrated that treatment of larval zebrafish with VitD analogs (e.g., alfacalcidiol) promoted the formation of mineralized bone, indicated by increased *cyp24a1* membrane-bound mGFP expression [72], which correlated with our findings (Supplementary Figure S3).

In summary, we demonstrated that the ability of high-dose VitD to significantly decrease fat accumulation in zebrafish livers was as effective as that of a calorie restriction diet, thereby preventing NAFLD progression. Moreover, the transcriptomic analysis highlighted five significant genes and revealed several metabolic pathways that might be important targets for NAFLD treatment. Our results indicated that the zebrafish is an attractive model to study the regulatory mechanisms involved in NAFLD. More importantly, these results might influence the manner in which NAFLD patients with COVID-19 should be treated, which is in accordance with the recommendations for VitD supplementation in selected populations of NAFLD patients [25]. It is possible that high VitD doses might reduce COVID-19 severity and/or mortality in NAFLD patients, while dosage should be regulated to prevent hypervitaminosis D [73].

## Data Availability

The data presented in this study are available on request from the corresponding author.

## Supplementary Materials

The following supporting information can be downloaded at: https://www.mdpi.com/article/10.3390/nu15061362/s1, Figure S1: Histological characterization of the various Zebrafish NAFLD models; Figure S2: BMI and fat levels in adult NAFLD zebrafish liver after feeding; Figure S3: Relative expression levels of *cyp24a1* in zebrafish liver as measured by RT-qPCR, and Figure S4: Metascape pathway analysis of top differentially expressed genes of NAFLD Zebrafish following high VitD diet compared to NAFLD untreated control. Table S1: Differentially and significantly expressed genes following transcriptomic analysis of NAFLD treated with high VitD compared to NAFLD untreated control; Table S2: Differentially expressed genes by RNA-sequencing, and Table S3: Top pathways differentially expressed in NAFLD supplemented with high VitD compared to non-VitD supplemented zebrafish.
