# Supplementary material for: Beneficial Effect of Vitamin D on Non-Alcoholic Fatty Liver Disease (NAFLD) Progression in the Zebrafish Model
Source: Nutrients. 2023 Mar 10;15(6):1362. doi: 10.3390/nu15061362 (PMC10052639; doi:10.3390/nu15061362)
Supplement: Supplementary file 1 [file nutrients-15-01362-s001.zip › nutrients-2242575-supplementary.pdf]

Beneficial effect of vitamin D on non-alcoholic fatty liver disease (NAFLD) progression in the zebrafish model

Lihi Grinberg[1¶], Fadwa Dabbah Assadi[1¶], Gideon Baum[1], Romy Zemel[2], Ran Tur-Kaspa[1,3], Chen Shochat[1], David Karasik[1&*], Marcela V Karpuj[1,4&*]

[1] Azrieli Faculty of Medicine, Bar-Ilan University, Safed, Israel

[2] Felsenstein Medical Research Center, Petah Tikva, Israel.

[3] Liver Institute, Rabin Medical Center, Petah Tikva, Israel

[4] Department of Biotechnology Engineering, Braude College, Karmiel, Israel.

Supplementary Figures and Tables

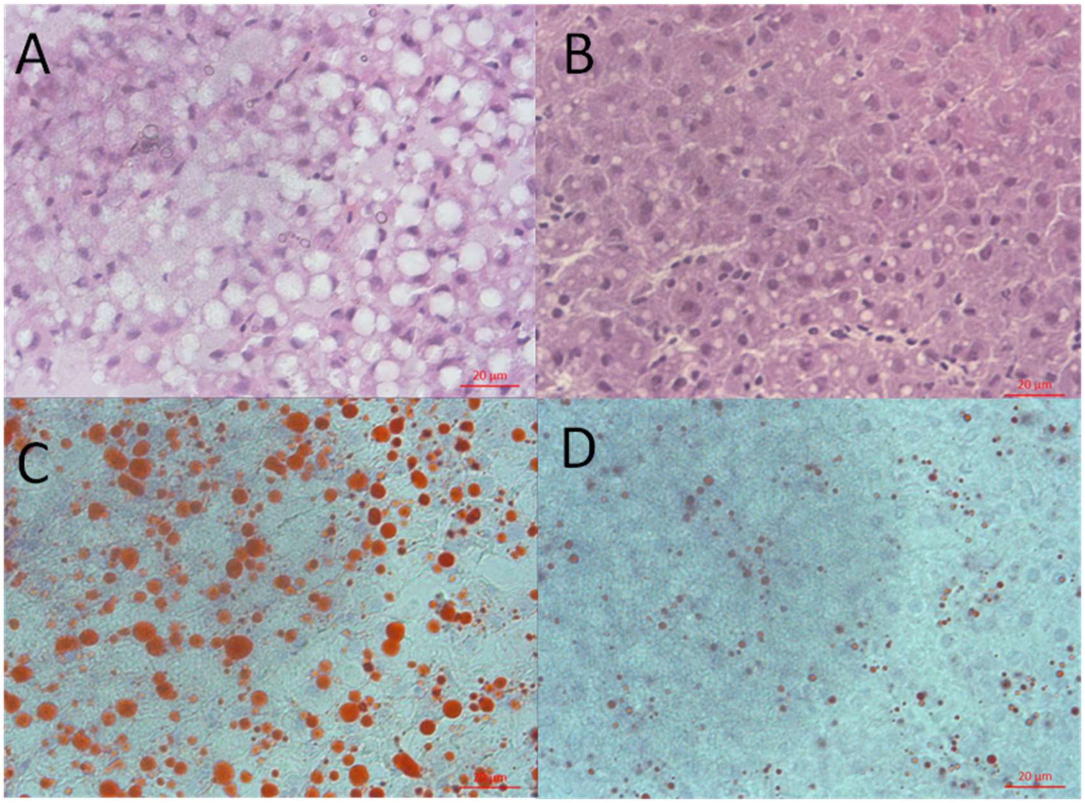

Supplementary Figure S1. Histological characterization of the various Zebrafish NAFLD model. Representative images of liver histological sections of adult zebrafish after 3 months of overfeeding. (A) overfed liver stained with hematoxylin and eosin (H&E), (B) control liver stained with H&E, (C) overfed liver stained with Oil Red O (ORO), (D) control liver stained with ORO. Magnification x40, Scale bar 20 µm.

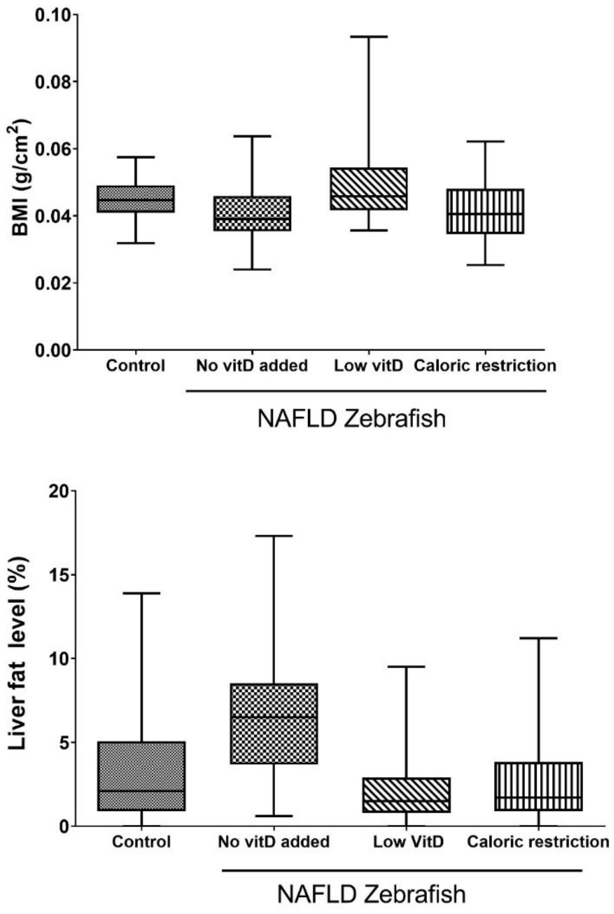

Supplementary Figure S2. BMI and fat levels in adult NAFLD zebrafish liver after feeding with: no VitD added (3 meal/day/3L tank), low VitD (3 meal/day/3L tank with 0.049 µg VitD per meal), calorie restriction diet (1 meal/day/3L tank). Control (2 meal/day/3L). NAFLD zebrafish with no VitD added had higher level of fat compared with other groups. When NAFLD zebrafish were treated with either VitD or caloric restriction diet, fat level dropped to control level.

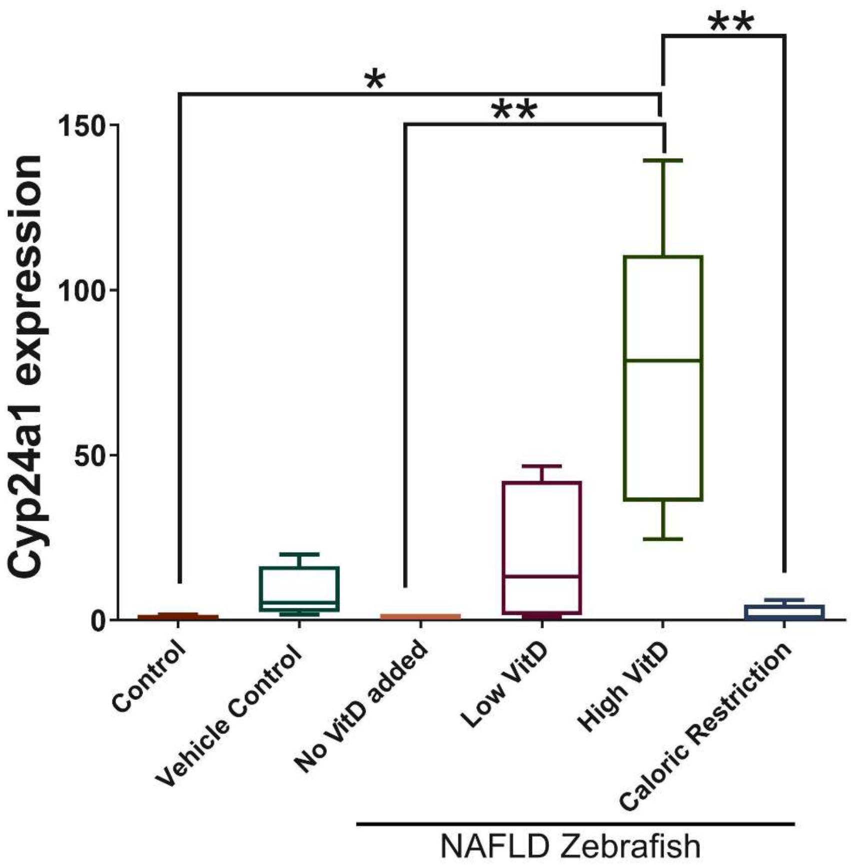

Supplementary Figure S3. Relative expression levels of *cyp24a1* (a gene coding the VitD catalyzing enzyme) in zebrafish liver as measured by RT-qPCR. Control and vehicle control groups were fed 2 times a day, and the same amount of ethanol used to dissolve VitD was added only to vehicle control group. NAFLD zebrafish were fed 3 times a day fish food with no VitD added, or low VitD (0.049 μg meal/day/3L tank), or high VitD (1.25 μg meal/day/3L tank). Caloric restriction group was fed fish food once a day. * $p<0.05$, ** $p < 0.01$.

Gene	log_2(fold_change)	p_value	q_value
dio3b	7.1	0.0005	0.03
**cyp24a1**	6.5	0.0001	0.01
gatm	5.9	0.0001	0.01
aqp9b	5.4	0.0002	0.01
igfbp1b	5.0	0.0001	0.01
zgc:171470	4.8	0.0001	0.01
gch2	4.2	0.0001	0.01
raver2	4.0	0.0001	0.01
mylipb	4.0	0.0001	0.01
ace	4.0	0.0001	0.01
LOC100149379	3.9	0.0002	0.01
cyp7a1a	3.9	0.0001	0.01
zgc:92137	3.9	0.0001	0.01
abcb11a	3.8	0.0001	0.01
hmgcra	3.6	0.0001	0.01
rgs2	3.5	0.0001	0.01
tbc1d32	3.5	0.0006	0.04
si:ch211-106h4.4	3.5	0.0001	0.01
LOC103910140	3.4	0.0001	0.01
sqlea	3.3	0.0001	0.01
slc16a12b	3.3	0.0004	0.03
LOC565702	3.3	0.0001	0.01
slc7a10a	3.3	0.0001	0.01
pltp	3.2	0.0001	0.01
adamts1	3.2	0.0001	0.01
si:ch73-330k17.3	3.2	0.0001	0.01

lss	3.1	0.0001	0.01
cited2	3.1	0.0001	0.01
LOC100536844	3.1	0.0001	0.01
ela2	3.1	0.0001	0.01
c6ast3	3.0	0.0001	0.01
LOC571431	3.0	0.0001	0.01
vtg4	3.0	0.0002	0.01
LOC557756	3.0	0.0001	0.01
LOC795897	2.9	0.0001	0.01
mvda	2.9	0.0002	0.02
si:ch211-222m15.19	2.9	0.0001	0.01
apoa4a	2.9	0.0001	0.01
sult1st1	2.9	0.0001	0.01
zgc:92745	2.8	0.0001	0.01
zgc:112368	2.8	0.0001	0.01
LOC100330331	2.8	0.0005	0.03
slc30a10	2.8	0.0001	0.01
irg1	2.8	0.0002	0.01
LOC100537376	2.8	0.0002	0.02
cpo	2.7	0.0001	0.01
itln3	2.7	0.0001	0.01
egln3	2.6	0.0001	0.01
alp3	2.6	0.0001	0.01
vtg1	2.6	0.0001	0.01
acsl1b	2.6	0.0001	0.01
ddit4	2.6	0.0001	0.01
LOC103910098	2.6	0.0001	0.01
p4ha1b	2.6	0.0001	0.01
LOC100536225	2.5	0.0001	0.01

grn1	2.5	0.0001	0.01
eno1a	2.5	0.0001	0.01
ctrb1	2.5	0.0001	0.01
si:dkey-57c15.4	2.5	0.0001	0.01
si:ch73-103b2.3	2.5	0.0004	0.03
zgc:112160	2.5	0.0001	0.01
LOC568900	2.5	0.0001	0.01
si:ch73-40a17.3	2.5	0.0001	0.01
zgc:66382	2.5	0.0004	0.03
si:dkey-14d8.6	2.5	0.0001	0.01
zgc:92041	2.4	0.0004	0.03
lygl1	2.4	0.0008	0.05
LOC100334171	2.4	0.0001	0.01
dnase1	2.4	0.0001	0.01
ebp	2.4	0.0001	0.01
pdzd3b	2.4	0.0001	0.01
si:dkey-14d8.7	2.4	0.0001	0.01
angptl2b	2.4	0.0001	0.01
ela2l	2.3	0.0003	0.02
si:ch211-240l19.6	2.3	0.0003	0.02
LOC553228	2.3	0.0001	0.01
LOC101886139	2.3	0.0001	0.01
si:dkey-286j15.1	2.3	0.0001	0.01
npc1l1	2.3	0.0001	0.01
pglyrp6	2.2	0.0001	0.01
si:ch211-240l19.5	2.2	0.0001	0.01
dhcr7	2.2	0.0001	0.01
lgals3bpb	2.2	0.0001	0.01
cpa1	2.2	0.0001	0.01

si:ch211-240l19.8	2.2	0.0004	0.03
asah2	2.2	0.0001	0.01
LOC100535194	2.2	0.0007	0.04
sycn	2.2	0.0001	0.01
ttc27	2.1	0.0004	0.03
LOC797571	2.1	0.0007	0.04
abca1b	2.1	0.0001	0.01
LOC101883346	2.1	0.0001	0.01
kpna2	2.1	0.0004	0.03
mpv17l2	2.1	0.0005	0.03
si:busm1-64d20.2	2.1	0.0001	0.01
fdft1	2.1	0.0001	0.01
fam151a	2.1	0.0001	0.01
LOC101883241	2.1	0.0001	0.01
amy2a	2.0	0.0001	0.01
osgn1	2.0	0.0001	0.01
cry-dash	2.0	0.0004	0.03
fdps	2.0	0.0001	0.01
zgc:153968	2.0	0.0003	0.02
rdh12	2.0	0.0002	0.01
ero1l	2.0	0.0001	0.01
LOC569007	1.9	0.0004	0.03
LOC559107	1.9	0.0001	0.01
fep15	1.9	0.0008	0.05
ctrl	1.9	0.0008	0.05
slc15a1a	1.9	0.0003	0.02
cry3	1.9	0.0001	0.01
stoml3b	1.9	0.0004	0.03
pyyb	1.9	0.0001	0.01

slc22a21	1.9	0.0001	0.01
cyp51	1.9	0.0003	0.02
bhlhe41	1.8	0.0001	0.01
slc5a8l	1.8	0.0001	0.01
ano5a	1.8	0.0001	0.01
si:dkey-30j10.5	1.8	0.0004	0.03
LOC100331289	1.8	0.0003	0.02
slc16a10	1.8	0.0008	0.05
sepp1b	1.8	0.0008	0.05
endou	1.8	0.0003	0.02
LOC100001887	1.8	0.0001	0.01
h2afvb	1.8	0.0004	0.03
LOC100331208	1.7	0.0001	0.01
sc5d	1.7	0.0001	0.01
slc39a4	1.7	0.0006	0.04
timp2a	1.7	0.0003	0.02
si:dkeyp-73d8.9	1.7	0.0004	0.03
cftr	1.7	0.0003	0.02
fabp7b	1.7	0.0005	0.03
mid1ip1l	1.7	0.0006	0.04
zgc:165502	1.7	0.0002	0.02
slc22a4	1.6	0.0003	0.02
zgc:86599	1.6	0.0004	0.03
zgc:66337	1.6	0.0002	0.01
slc7a3a	1.6	0.0007	0.04
wu:fb55g09	1.6	0.0002	0.02
zgc:66484	1.6	0.0006	0.04
alpi.2	1.6	0.0005	0.03
srebf2	1.6	0.0004	0.03

LOC101883180	1.6	0.0002	0.02
cox6a1	1.6	0.0002	0.02
aqp8a.2	1.6	0.0005	0.03
ilf2	1.5	0.0006	0.04
cpox	1.5	0.0005	0.03
klb	1.5	0.0008	0.05
btr07	1.5	0.0008	0.05
aldh4a1	-1.4	0.0007	0.04
pex3	-1.5	0.0008	0.05
crot	-1.5	0.0007	0.04
ddt	-1.5	0.0006	0.04
acad8	-1.6	0.0006	0.04
nr2f2	-1.6	0.0004	0.03
LOC100331428	-1.6	0.0007	0.04
hspb8	-1.6	0.0006	0.04
cyp27a7	-1.6	0.0003	0.02
ptgdsa	-1.6	0.0003	0.02
slc12a4	-1.6	0.0002	0.01
zgc:193682	-1.6	0.0003	0.02
zgc:171500	-1.6	0.0008	0.05
ppp1r3cb	-1.7	0.0002	0.02
adrb2b	-1.7	0.0005	0.03
nid1a	-1.7	0.0004	0.03
ptplb	-1.7	0.0005	0.03
xdh	-1.7	0.0002	0.02
msrb1a	-1.7	0.0001	0.01
zgc:110783	-1.7	0.0002	0.02
LOC103912051	-1.7	0.0004	0.03
zgc:103678	-1.7	0.0003	0.02

zgc:195173	-1.7	0.0004	0.03
LOC103911503	-1.7	0.0001	0.01
slco1d1	-1.8	0.0007	0.04
si:dkey-58f10.11	-1.8	0.0001	0.01
hgd	-1.8	0.0007	0.04
zmp:0000000758	-1.8	0.0001	0.01
zgc:92040	-1.8	0.0003	0.02
slc43a1b	-1.8	0.0002	0.01
si:ch211-188c16.1	-1.8	0.0002	0.02
LOC100002960	-1.8	0.0005	0.03
gys2	-1.8	0.0002	0.01
amdhd1	-1.8	0.0001	0.01
igf2b	-1.9	0.0002	0.01
urad	-1.9	0.0001	0.01
LOC100537915	-1.9	0.0004	0.03
LOC100330440	-1.9	0.0001	0.01
sirt3	-1.9	0.0005	0.03
pxmp2	-1.9	0.0001	0.01
bnip3	-1.9	0.0001	0.01
ces2	-1.9	0.0008	0.05
tldc1	-1.9	0.0002	0.02
si:dkey-65b13.9	-1.9	0.0006	0.04
zgc:153031	-1.9	0.0001	0.01
LOC100332592	-1.9	0.0006	0.04
map4k2	-1.9	0.0002	0.01
foxa2	-2.0	0.0001	0.01
zgc:165582	-2.0	0.0001	0.01
h1fx	-2.0	0.0001	0.01
LOC797311	-2.0	0.0003	0.02

irg1l	-2.0	0.0002	0.02
si:dkeyp-89c11.2	-2.0	0.0002	0.01
robo2	-2.0	0.0002	0.01
slco2a1	-2.0	0.0001	0.01
cetp	-2.0	0.0003	0.02
si:ch211-222e20.4	-2.0	0.0004	0.03
abcb11b	-2.0	0.0002	0.01
adka	-2.0	0.0001	0.01
tfcp2l1	-2.0	0.0005	0.03
LOC100330415	-2.0	0.0002	0.01
acsbg1	-2.1	0.0004	0.03
zbtb20	-2.1	0.0004	0.03
cat	-2.1	0.0005	0.03
slc25a32a	-2.1	0.0001	0.01
adh8a	-2.1	0.0006	0.04
si:dkey-9i23.8	-2.1	0.0003	0.02
ppp1r3b	-2.1	0.0004	0.03
si:busm1-266f07.2	-2.1	0.0001	0.01
igf2a	-2.1	0.0001	0.01
pls3	-2.1	0.0001	0.01
krt91	-2.1	0.0003	0.02
agpat9	-2.1	0.0001	0.01
slc16a13	-2.1	0.0002	0.01
angptl3	-2.1	0.0007	0.04
zgc:112315	-2.1	0.0001	0.01
bag3	-2.1	0.0001	0.01
slc43a3b	-2.2	0.0003	0.02
inpp5f	-2.2	0.0001	0.01
rnasel3	-2.2	0.0006	0.04

bcat2	-2.2	0.0007	0.04
histh1l	-2.2	0.0001	0.01
mbnl3	-2.2	0.0001	0.01
LOC566131	-2.2	0.0005	0.03
rbp2b	-2.2	0.0001	0.01
alas2	-2.2	0.0001	0.01
ugt5e1	-2.2	0.0005	0.03
rwdd	-2.3	0.0006	0.04
slc25a36a	-2.3	0.0001	0.01
LOC100535291	-2.3	0.0001	0.01
si:ch211-276k2.2	-2.3	0.0007	0.04
dpys	-2.3	0.0001	0.01
miox	-2.4	0.0001	0.01
cmpk2	-2.4	0.0003	0.02
urah	-2.4	0.0001	0.01
ccrn4la	-2.4	0.0001	0.01
pbld1	-2.4	0.0001	0.01
mgea5	-2.4	0.0001	0.01
ate1	-2.4	0.0007	0.04
col5a1	-2.4	0.0002	0.01
hbaa1	-2.4	0.0005	0.03
LOC103911768	-2.4	0.0004	0.03
cahz	-2.4	0.0001	0.01
ba1l	-2.4	0.0002	0.01
zgc:123275	-2.4	0.0001	0.01
msrb2	-2.4	0.0001	0.01
cyp39a1	-2.4	0.0001	0.01
zmp:0000001078	-2.4	0.0001	0.01
LOC103909258	-2.5	0.0001	0.01

rsad2	-2.5	0.0001	0.01
slc4a1a	-2.5	0.0003	0.02
LOC100332418	-2.5	0.0001	0.01
zgc:161973	-2.6	0.0001	0.01
rnd1l	-2.6	0.0001	0.01
atp2a1l	-2.6	0.0007	0.04
epb41b	-2.6	0.0001	0.01
pebp1	-2.6	0.0001	0.01
abhd3	-2.6	0.0002	0.02
ces3	-2.6	0.0001	0.01
mpx	-2.7	0.0001	0.01
wu:fd44f11	-2.7	0.0001	0.01
LOC100535383	-2.7	0.0001	0.01
LOC101887004	-2.7	0.0005	0.03
si:dkey-71h2.2	-2.7	0.0002	0.01
camk2n1a	-2.7	0.0001	0.01
me1	-2.7	0.0001	0.01
LOC100332947	-2.7	0.0001	0.01
LOC101886683	-2.7	0.0006	0.04
nr0b1	-2.7	0.0001	0.01
sult2st3	-2.7	0.0004	0.03
cry2a	-2.7	0.0007	0.04
LOC100537842	-2.7	0.0001	0.01
LOC565454	-2.8	0.0005	0.03
LOC793037	-2.9	0.0001	0.01
ppdpfb	-2.9	0.0001	0.01
cyp8b3	-3.0	0.0008	0.05
zgc:194626	-3.0	0.0006	0.04
ba1	-3.0	0.0001	0.01

cldn11a	-3.1	0.0006	0.04
si:ch211-171h4.3	-3.1	0.0001	0.01
cpeb4	-3.2	0.0001	0.01
ypel3	-3.2	0.0001	0.01
wu:fi74c02	-3.2	0.0001	0.01
zgc:86725	-3.2	0.0001	0.01
LOC101883275	-3.2	0.0001	0.01
cyp2ae1	-3.2	0.0003	0.02
igfbp2b	-3.2	0.0001	0.01
sc:d0202	-3.2	0.0001	0.01
lpin1	-3.2	0.0005	0.03
tor3a	-3.2	0.0001	0.01
mfsd2ab	-3.3	0.0001	0.01
wu:fi27h03	-3.3	0.0005	0.03
spp2	-3.3	0.0001	0.01
LOC100538158	-3.3	0.0001	0.01
tubb5	-3.4	0.0001	0.01
c1qtnf1	-3.4	0.0001	0.01
acta1a	-3.4	0.0001	0.01
Lipg	-3.5	0.0004	0.03
zgc:171534	-3.5	0.0001	0.01
cry2a	-3.6	0.0001	0.01
zgc:92880	-3.7	0.0001	0.01
si:dkey-22f5.9	-3.7	0.0001	0.01
trmt10b	-3.8	0.0002	0.01
spam1	-3.9	0.0001	0.01
mhc2dab	-3.9	0.0001	0.01
si:ch211-175f11.5	-4.0	0.0001	0.01
ggact.1	-4.1	0.0002	0.02

si:ch211-5k11.6	-4.2	0.0001	0.01
elovl6	-4.2	0.0001	0.01
zgc:73273	-4.2	0.0001	0.01
si:dkey-18p12.4	-4.3	0.0001	0.01
Mia	-4.3	0.0001	0.01
krt5	-4.4	0.0001	0.01
aldh1l1	-4.5	0.0001	0.01
slc12a10.3	-4.8	0.0001	0.01
LOC567192	-4.8	0.0001	0.01
dr1	-5.0	0.0001	0.01
si:busm1-160c18.6	-5.0	0.0001	0.01
hsc70	-5.2	0.0001	0.01
dio2	-5.2	0.0001	0.01
LOC100329520	-5.7	0.0007	0.04
igf1	-5.7	0.0001	0.01
zgc:172271	-5.9	0.0001	0.01

Supplementary Table S1. Differentially and significantly expressed genes following transcriptomic analysis of NAFLD treated with high VitD compared to NAFLD untreated control.

Gene	Primer Sequence (5'-3')
ef1α (eef1a1l1)	5'-CGTCTGCCACTTCAGCATGTG-3'
	5'- ACTTGCAGGCGATGTGAGCAG-3'
adh8a	5'-CGAGTACACCGTCATCAAC-3'
	5'-AGCACCGAGTCCGAATAC-3'
ddt	5'-GTGGTAAAGCCTGACCTGCC-3'
	5'-TGTGATGGCTCCAGAGGGTAA-3'
rbp2b	5'-CAACAGGGCATTGAAGACTCTGG-3'
	5'- TCCCCGATTGGCTTTCTCACCC-3'
histh1l	5'-CTACCTCTGTACCTGATACACAAA-3'
	5'-GGACATATTGGACACTTTCC-3'
apoc2	5'- AAGGGAACCATCGCTGTTGT
	5'- GCTGGTCTTGAAAGATGCCG
h1fx	5'- AAAACGGCTCGTCGCTTTTC-3'
	5'- TTGGCTCCGAATCCCTTCAC-3'
dio2	5'- GGATGAGTCGGAAAGGTGAA-3'
	5'- CCACACTAAGCAAGCCCATT-3'
aldh1a1	5'- GGACTGGACACAGGACCAAT-3'
	5'- AGGAAGCGCTTGTAGATGCT-3'
apoc4	5'- TGCTTGACCTTGTCCAACCC-3'
	5'- TAACCAGCCACTGTCTTTGCC-3'
cyp24a	5'-AAAAGTCAACGGCAAAATGG-3'
	5'-GTGTGGTCCTTCCACGTCTT-3'
ces1	5'- ATACCCTCCATCAAAGTTGCGT-3'
	5'- TGTAACTTTCACCTGACCATCCC-3'

Supplementary Table S2. Differentially expressed genes by RNA-sequencing between (A) NAFLD untreated and treated with high VitD and NAFLD and vehicle-control groups

[Molecular Functions]

	ID	Name	pValue	FDR B&H	FDR B&Y	Bonferroni
1	GO:0005344	oxygen transporter activity	0.000002	0.0003	0.002	0.0003
2	GO:0031721	hemoglobin alpha binding	0.000008	0.0008	0.005	0.002
3	GO:0030492	hemoglobin binding	0.00008	0.004	0.02	0.02
4	GO:0019825	oxygen binding	0.0001	0.004	0.02	0.02
5	GO:0005506	iron ion binding	0.0002	0.006	0.03	0.03
6	GO:0016491	oxidoreductase activity	0.0002	0.007	0.04	0.04

[GO: Biological Process]

	ID	Name	pValue	FDR B&H	FDR B&Y	Bonferroni
1	GO:0015671	oxygen transport	0.000002	0.002	0.01	0.002
2	GO:0055114	oxidation-reduction process	0.000004	0.002	0.01	0.004
3	GO:0019752	carboxylic acid metabolic process	0.000004	0.002	0.01	0.005
4	GO:0032787	monocarboxylic acid metabolic process	0.000007	0.002	0.01	0.008
5	GO:0015669	gas transport	0.000008	0.002	0.01	0.009
6	GO:0043436	oxoacid metabolic process	0.00001	0.002	0.01	0.012
7	GO:0044712	single-organism catabolic process	0.00001	0.002	0.01	0.012

[Pathways: BioSystems/REACTOME]

	ID	Name	pValue	FDR B&H	FDR B&Y	Bonferroni
1	1270190	Phase 1 - Functionalization of compounds	0.0001	0.01	0.07	0.03
2	1269630	Signaling by Retinoic Acid	0.0001	0.01	0.07	0.04
3	1270002	Lipid digestion, mobilization, and transport	0.0001	0.01	0.07	0.04
4	1270189	Biological oxidation	0.0002	0.01	0.07	0.05

[Gene Families (acc. to genenames.org)]

	ID	Name	pValue	FDR B&H	FDR B&Y	Bonferroni

1	940	Hemoglobin subunits	0.0000002	0.000003	0.000009	0.000003
2	461	Carboxylesterases	0.00001	0.0001	0.0003	0.0002
3	550	Fatty acid binding protein family	0.0002	0.0008	0.003	0.002
4	405	Apolipoproteins	0.0003	0.001	0.004	0.004
5	864	Histones	0.0004	0.001	0.004	0.005
6	591	CD molecules|C1-set domain containing	0.001	0.003	0.01	0.02

Supplementary Table S3. Top pathways differentially expressed in NAFLD supplemented with high VitD compared to non-VitD supplemented zebrafish. Significant according to Bonferroni-adjusted p-value (<0.05). B&H - Benjamini and Hochberg; B&Y - Benjamini and Yekutieli's FDR

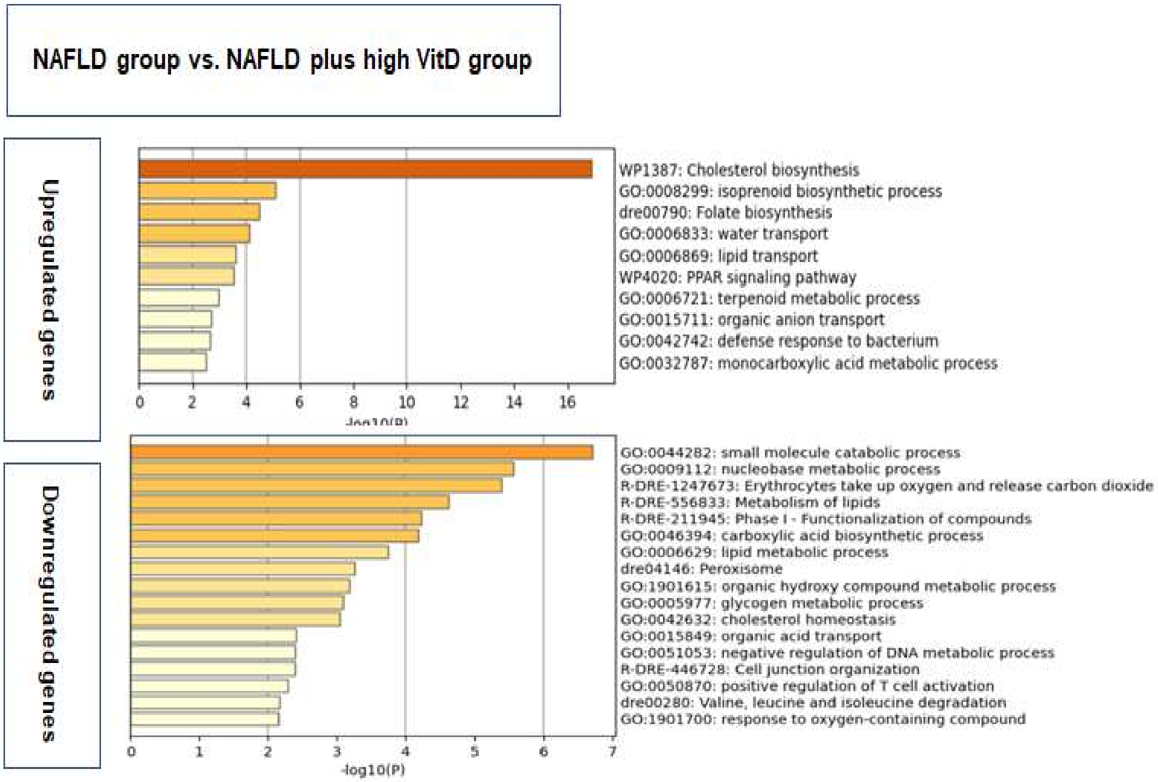

Supplementary Figure S4. Metascape pathway analysis [39] of top differentially expressed genes (listed in supplementary table 1) of NAFLD Zebrafish following high VitD diet compared to NAFLD untreated control.
